# Aberrant functional connectivity and the intrinsic activity of primary visual network and their relationship with adolescent atypical depression symptoms

**DOI:** 10.3389/fpsyt.2025.1690871

**Published:** 2025-12-08

**Authors:** Yaoyao Li, Xiaoyan Liu, Hanying Liu, Bing Tang, Jize Xie, Chunhe Ma, Yingyi Yu, Sha Wu, Yi Li

**Affiliations:** 1 The Second Ward of Psychosomatic Department, Affiliated Mental Health Center of ZheJiang University School of medicine and HangZhou Seventh People`s Hospital, Hangzhou, China; 2 The Fifth Ward of Psychiatry Department, Affiliated Mental Health Center of ZheJiang University School of Medicine and HangZhou Seventh People’s Hospital, Hangzhou, China; 3 The Department of Psychiatry, QuZhou Third People’s Hospital, Quzhou, China; 4 The Second Clinical College, Zhejiang Chinese Medical University, Hangzhou, China; 5 The First Ward of Psychiatry Department, Zhejiang West Branch of Hangzhou Seventh People’s Hospital, Hangzhou, China; 6 The Second Ward of Psychiatry Department, Zhejiang West Branch of Hangzhou Seventh People’s Hospital, Hangzhou, China; 7 The Department of Radiology, Affiliated Mental Health Center of ZheJiang University School of Medicine and HangZhou Seventh People`s Hospital, Hangzhou, China; 8 The Department of Intensive Care Unit, Affiliated Mental Health Center of ZheJiang University School Of Medicine and HangZhou Seventh People`s Hospital, Hangzhou, China

**Keywords:** atypical depression, resting-state fMRI, functional connectivity, independent component analysis, primary visual network, fusiform face area

## Abstract

**Background:**

The pathophysiological mechanisms of adolescent atypical depression (A-D) remain unclear. This study used resting-state functional magnetic resonance imaging (rs-fMRI) and independent component analysis (ICA) to compare functional network connectivity (FNC) and intrinsic brain activity between healthy control, A-D, and non-atypical depression (N-AD) adolescents.

**Methods:**

Fifteen healthy control (HC), 17 A-D, and 18 N-AD adolescents underwent rs-fMRI. Clinical symptoms were assessed using the 30-item Inventory of Depressive Symptomatology (IDS-30R). ICA was employed to analyze internetwork FNC and fractional amplitude of low-frequency fluctuations (fALFF).

**Results:**

The A-D group exhibited decreased connectivity between the primary visual (IC4) and visuospatial (IC10) networks but increased connectivity between IC4 and the sensorimotor network (IC11), and between the ventral and dorsal default mode networks (IC8 and IC17, respectively). Higher fALFF values were found in the right fusiform face area and left superior occipital gyrus of the A-D group than the N-AD group. Increased appetite correlated positively with IC4–IC10 connectivity (*r* = 0.573, *p* = 0.016), and interpersonal rejection sensitivity was correlated with fALFF in the right fusiform area (*r* = 0.625, *p* = 0.007).

**Conclusion:**

Aberrant functional connectivity of the visual network and altered activity in visual-processing regions are associated with specific A-D symptoms, providing new insights into its neurophysiology.

## Introduction

Depression exhibits high clinical heterogeneity and is divided into various subtypes. Atypical depression (A-D), recognized as a distinct subtype of major depressive disorder (MDD) in the *Diagnostic and Statistical Manual of Mental Disorders*, Fifth Edition (DSM-5) (1), is characterized by hypersomnia, increased appetite/weight gain, leaden paralysis, and heightened sensitivity to interpersonal rejection, as well as the typical symptoms of MDD like depressed mood, loss of interest, and lack of energy (2). A multicenter study in China reported a prevalence of A-D at 15.3% in all depression cases (3). Epidemiological and clinical studies from Western countries have indicated that the prevalence of A-D among individuals with depression ranges from 15.7% to 36.4% (4, 5). Furthermore, depressed patients with atypical features demonstrate more severe clinical manifestations compared to those without atypical features, including an earlier age of onset, a greater tendency toward chronicity, a higher risk of conversion to bipolar disorder II (BP-II), a higher prevalence in female individuals, and potentially greater impairment and associated risks (6–10). Given the trend of depression onset occurring at younger ages, A-D represents a subtype requiring significant attention in the treatment of adolescent MDD.

Since the concept of “atypicality” was proposed in the 1960s, clinicians have noted that A-D not only presents distinct clinical features compared to other forms of depression—such as sleep disturbances, appetite/weight changes, leaden paralysis, and interpersonal rejection sensitivity—but also shows greater responsiveness to monoamine oxidase inhibitors (MAOIs) (11, 12). Moreover, as research progressed, a retrospective analysis by Juruena et al. (13) on hypothalamic-pituitary-adrenal (HPA) axis function in A-D indicated lower HPA axis activity in A-D patients compared to melancholic depression patients. Another systematic review (14) demonstrated an association between A-D and metabolic syndrome, a link not observed in other depressive subtypes. An electroencephalography (EEG) study (15) revealed that A-D patients exhibit distinct hemispheric differences in processing chimeric faces compared to patients with other depression subtypes and healthy controls. Specifically, A-D patients showed significantly higher right hemisphere activity, a phenomenon independent of gender, comorbid anxiety disorders, vegetative symptoms, and self-rated depression severity. These studies have collectively indicated significant differences between A-D and other depressive subtypes. However, despite the concept existing for decades, the pathophysiological mechanisms underlying A-D remain largely unknown, particularly regarding the mechanisms differentiating its symptoms from those of typical depression. Therefore, exploring the neurobiological and psychopathological characteristics of A-D from new perspectives is essential.

The primary visual network is a crucial region in the human brain for analyzing, encoding, and transmitting visual information. It also contributes to the psychopathology and cognitive deficits observed in MDD (16). Research by Song et al. indicates that the functional connectivity (FC) between the primary visual network and the motor network is correlated with the severity of psychomotor retardation in MDD patients (17). Another study found that MDD patients exhibit reduced FC between the tail of the hippocampus and the primary visual cortex—an abnormality that can be normalized following electroconvulsive therapy (ECT) (18). Even in remitted MDD patients, who are more likely than never-depressed individuals to show difficulties in inhibiting attention to emotional distractors, these impairments are associated with primary visual cortex function (19). Together, these findings highlight the role of abnormal primary visual network function in MDD. However, to date, little is known about its implications in adolescent A-D.

Resting-state functional magnetic resonance imaging (rs-fMRI) is a technique that maps the brain in a non-task state by detecting spontaneous fluctuations in the blood oxygen level-dependent (BOLD) signal, reflecting intrinsic brain activity (20, 21). This method reveals how the brain integrates neural signals from internal and external environments, thereby enhancing our understanding of cognitive and emotional processes. Due to its non-invasiveness and high temporal resolution, rs-fMRI has become a vital tool for studying neurological and psychiatric disorders. It is widely used to explore potential neural mechanisms and identify potential biomarkers for these conditions (22–24). Particularly in depression research, rs-fMRI has uncovered possible neurobiological mechanisms (25–27) and has been applied to guide clinical treatments [e.g., repetitive transcranial magnetic stimulation (rTMS)] (28). It is a clinically validated and reliable method for investigating the neurobiological underpinnings of psychiatric disorders.

However, studies using rs-fMRI to investigate A-D are relatively scarce, and even fewer studies have focused on the adolescent population. Independent component analysis (ICA) is a data-driven rs-fMRI analysis method independent of predefined seed regions. It effectively mitigates the variability in results stemming from seed selection differences and significantly reduces noise effects (29). Furthermore, by utilizing predefined resting-state network templates (30), ICA facilitates the analysis of FC between networks, making it a mature and effective rs-fMRI analysis technique. This study aimed to employ rs-fMRI and the data-driven approach of ICA (31) to investigate differences in functional network connectivity (FNC) among adolescents with A-D and adolescents with non-atypical depression (N-AD). We assessed patients’ clinical characteristics using relevant scales. We conducted comparative analyses of functional connectivity and its correlation with clinical scale scores across the three groups: healthy control (HC), A-D, and N-AD. We hypothesized that A-D patients exhibit distinct patterns of functional connectivity between brain networks compared to HC and N-AD patients and that these abnormalities correlate with clinical symptom severity. We believe that these findings will contribute to understanding the unique neurobiological mechanisms of A-D, which is helpful for developing personalized treatment strategies for this specific subtype.

## Materials and methods

### Participants and clinical assessment

This study complied with the tenets of the Declaration of Helsinki and was approved by the Ethics Committee of Hangzhou Seventh People’s Hospital (research no. 2023-021, 2025-039/040). It was also registered with the Chinese Clinical Trial Registry (Registration URL: https://www.medicalresearch.org.cn/clinicalResearch/researchInfo?id=87ff969a-0dc9-426f-8f0c-8f0df61343fe; Registration Number: MR-33-24-030536). Written informed consent was obtained from all participants and their legal guardians after a complete description of the study. Finally, 15 HCs (three male and 12 female individuals), 17 patients with A-D (two male and 15 female individuals), and 18 patients with N-AD (two male and 16 female individuals) who met the study criteria were recruited from the inpatient department of Hangzhou Seventh People’s Hospital from March 2023 to November 2023 and from April 2025 to September 2025.

The inclusion criteria for MDD included the following: 1) age 12–18 years, Han ethnicity, right-handed; 2) meets the DSM-5 diagnostic criteria for MDD, with a 17-item Hamilton Depression Rating Scale (HAMD-17) score >17 and a Bech–Rafaelsen Mania Rating Scale (BRMS) score <5; 3) understands the purpose and risks of the study and voluntarily participates; 4) ability to independently complete the clinical symptom assessments, scale evaluations, and fMRI scanning required for this study; 5) absence of family history of psychiatric disorders or neurological development disorders; and 6) for the A-D group, specifically meeting the diagnostic criteria for major depressive disorders with atypical features. Diagnoses were confirmed through structured clinical interviews conducted by two senior psychiatrists with extensive clinical experience. The inclusion criteria for HC included the following: 1) age 12–18 years, Han ethnicity, right-handed; 2) does not meet any diagnostic criteria of psychiatry disorders in the DSM-5, with a HAMD-17 score <7 and a BRMS score < 5; 3) understands the purpose and risks of the study and voluntarily participates; 4) ability to independently complete the clinical symptom assessments, scale evaluations, and fMRI scanning required for this study; and 5) absence of family history of psychiatric disorders or neurodevelopmental disorders.

The exclusion criteria included the following: 1) age below 12 years or above 18 years; 2) current or previous diagnosis of alcohol/substance dependence or other psychiatric disorders; 3) severe physical illness; 4) history of ECT within the past 6 months; 5) inability to complete clinical scale assessments; and 6) any contraindication to MRI or MRI images failing to meet quality requirements despite completion of the scan (translation > 1.5 mm, rotation > 1.5°). Demographic and clinical characteristic data, including neuropsychological scales and clinical features, are presented in **Table 1**.

**Table 1:** Results of demographic and behavioral scores.

Items	HC	A-D	N-AD	F/T/*χ*^2^ value	*p*-Value
Age	15.733 ± 1.944	15.591 ± 1.460	15.062 ± 1.626	0.770^a^	0.469
Gender (male/female)	3/12	2/15	2/16	0.644^*^	0.725
Education level (years)	8.533 ± 1.598	9.250 ± 1.342	8.944 ± 1.830	1.253^a^	0.295
Illness duration (months)	NA	26.688 ± 17.431	25.833 ± 18.950	0.136^b^	0.893
HAMD	NA	22.000 ± 3.579	23.556 ± 4.355	−1.108^b^	0.276
HAMA	NA	24.438 ± 2.220	23.111 ± 2.698	1.553^b^	0.130
Sleep disturbances	NA	1.882 ± 0.600	0.111 ± 0.323	10.955^b^	<0.001
Appetite changes	NA	1.177 ± 1.551	0.111 ± 0.583	2.661^b^	0.015
Weight changes	NA	1.118 ± 1.577	0.167 ± 0.383	2.420^b^	0.026
Interpersonal rejection sensitivity	NA	2.118 ± 0.601	0.056 ± 0.236	13.234^b^	<0.001

HC, healthy control; A-D, atypical depression; N-AD, non-atypical depression; HAMD, Hamilton Depression Rating Scale; HAMA, Hamilton Anxiety Rating Scale.

^*^This item was obtained by *χ*^2^ test.

a This result was obtained by ANOVA.

b This result was obtained by t-test.

### Clinical assessment

Both patient groups completed baseline assessments using the HAMD and Hamilton Anxiety Rating Scale (HAMA). The A-D group also completed the 30-item Inventory of Depressive Symptomatology (IDS-30R) (32). Scores from specific IDS-30R items (Item 4: Hypersomnia; Items 11/12: Appetite Change; Items 13/14: Weight Change; Item 29: Rejection Sensitivity) were used to assess the four core symptoms of the A-D group. In our statistical analysis, negative values were assigned to item 11 (decreased appetite) and positive values to item 12 (increased appetite) to create a unified appetite change score. The same polarity adjustment was applied to items 13 and 14.

### Functional magnetic resonance imaging data acquisition

The MRI data for all participants were acquired using a Siemens 3.0T MRI scanner (MAGNETOM Prisma, Erlangen, Germany) with a 64-channel head coil. Structural imaging: T1-weighted images were acquired using a magnetization-prepared rapid gradient-echo (T1-MPRAGE) sequence with the following parameters: repetition time (TR) = 3.7 ms, echo time (TE) = 2.2 ms, field of view (FOV) = 240 × 240 mm^2^, resolution = 1 × 1 × 1 mm^3^, 208 sagittal slices, slice gap = 0 mm, and acquisition time = 2 minutes 28 seconds.

Resting-state fMRI: BOLD images were acquired using an echo-planar imaging (EPI) sequence with the following parameters: TR = 800 ms, TE = 37 ms, FOV = 240 mm, resolution = 2 × 2 × 2 mm^3^, with no slice gap, 72 slices per volume oriented along the anterior–posterior commissure (AC-PC) line, 600 volumes acquired, and acquisition time = 8 minutes. Sound-attenuating headphones and foam padding were used to minimize head motion and reduce scanner noise. Participants were instructed to keep their eyes closed, remain relaxed and awake, and minimize movement during the resting-state scan.

### Functional magnetic resonance imaging data preprocessing

Resting-state fMRI data preprocessing was performed using fMRIPrep (33) (https://fmriprep.org/en/stable/). The preprocessing steps included the following: 1) converting DICOM format files to NIFTI format; 2) discarding the first 10 volumes to allow for signal stabilization; 3) slice timing correction; 4) realignment (motion correction), and in this step, the NeuroImaging & Surgical Technologies Lab (NIST) Pediatric template (4.5–18.5 years) was employed; 5) segmentation and spatial normalization to the Montreal Neurological Institute (MNI) standard space; and 6) spatial smoothing using a Gaussian kernel with a full-width at half maximum (FWHM) of 4 × 4 × 4 mm^3^.

### Independent Component Analysis

Group ICA was performed on the preprocessed data using the GIFT toolbox (Group ICA of fMRI Toolbox, V3.0B, https://trendscenter.org/software/, Georgia, USA). The specific procedure was as follows. Parameter setting: Preprocessed data from all subjects were imported. The number of independent components was estimated as 20 (34, 35). What must be paid attention to is that 20 of the independent components are very small, which may be helpful in obtaining large-scale, easily interpretable networks, but it may merge functional sub-networks, losing finer-grained information. Future studies with a higher number of components could explore these more granular networks. Data dimensionality was reduced using principal component analysis (PCA) in two stages (30 components retained in the first stage). The Infomax algorithm was used for ICA decomposition. The ICASSO approach was employed for group ICA, the ICA decomposition was repeated 20 times for stability, and the results were averaged.

#### Analysis execution

The parameter file (*parameter_info.mat) generated in the previous step was selected. The analysis steps were configured and run sequentially: Resume/Initialize Parameters, Group Data Reduction, Calculate ICA/IVA, Back-Reconstruct Components, Calibrate Components, and Group Stats. Back-reconstruction involved reconstructing individual subject component maps (spatial) and time courses for each component; then, Fisher’s transformation was used to transform correlations to z-scores, which can improve the normality.

#### Component identification

All components for all subjects were displayed using the Display GUI module within GIFT. Components were identified by comparing them to the resting-state network (RSN) templates provided within the GIFT toolbox and through visual inspection using the component labeler tool and manual observation. Components were matched to known RSNs based on the highest spatial correlation with template networks.

#### Component validation

Analysis of variance (ANOVA) and Tukey’s honestly significant difference (HSD) *post-hoc* analysis were performed on the IC4 map across all subjects (voxel-wise). Results were thresholded at false discovery rate (FDR)-corrected *p* < 0.05. The resulting statistically significant spatial maps were saved as masks for subsequent analysis.

#### Group comparison of network connectivity

ANOVA and Tukey’s HSD *post-hoc* analysis were performed to compare functional connectivity (component time course correlations) of each identified network between the two groups. Multiple comparisons correction was applied using FDR < 0.05. Cohen’s *f* was conducted to evaluate the effect size, and the equation of *Cohan’s f* (36).


$$f=\sqrt{\frac{\eta^{2}}{1-\eta^{2}}\;\;}$$

#### Regional analysis within networks

For networks showing significant between-group differences in connectivity, voxel-wise ANOVA and Tukey’s HSD *post-hoc* analysis were performed within the spatial extent of the corresponding network mask (saved in component validation) to identify specific brain regions contributing to the difference. The fractional amplitude of low-frequency fluctuations (fALFF) was computed using the DPABI software, according to Zou et al. (37) method and the DPABI manual. Multiple comparisons correction was applied using Gaussian random field (GRF) theory (voxel-level *p* < 0.001, cluster-level *p* < 0.05). Values from significantly different brain regions were extracted using the DPABI package for further statistical analysis.

### Statistical analysis

Descriptive statistics of demographic and clinical data were computed using SPSS (version 22.0). Pearson’s correlation analyses were conducted to examine the associations between intra-network functional connectivity (derived from ICA) and scores on the IDS-30R scales. Statistical significance was set at a threshold of *p* < 0.05. All figures were prepared using GraphPad Prism (version 9.5).

## Results

### Demographic and behavioral results

No significant differences were found among the three groups in terms of age, sex ratio, HAMD scores, HAMA scores, or duration of illness (for patient groups, see **Table 1**).

### Resting-state functional magnetic resonance imaging analysis results

#### Independent component analysis and component selection

**Figure 1:**
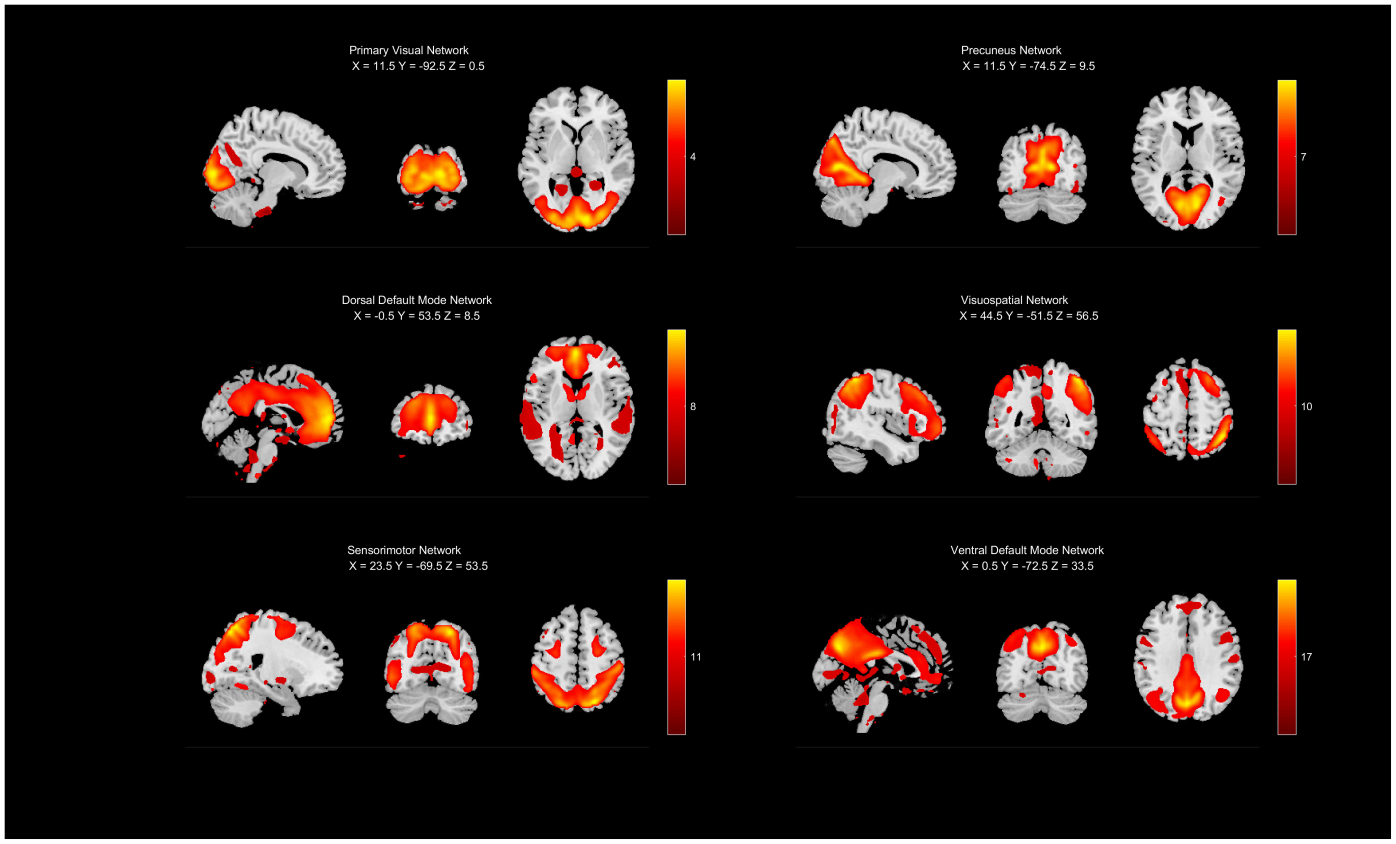
The selected ICA components of this study. ICA, independent component analysis.

Following comparison using the component labeler tool within the GIFT software and visual inspection, components exhibiting a spatial correlation coefficient greater than 0.2 with the template resting-state networks were selected. Six independent components (ICs) were ultimately identified and retained for further analysis: primary visual network (IC4), precuneus network (IC7), dorsal default mode network (IC8), visuospatial network (IC10), sensorimotor network (IC11), and ventral default mode network (IC17). These components are illustrated in **Figure 1**.

#### Functional network connectivity analysis

**Figure 2:**
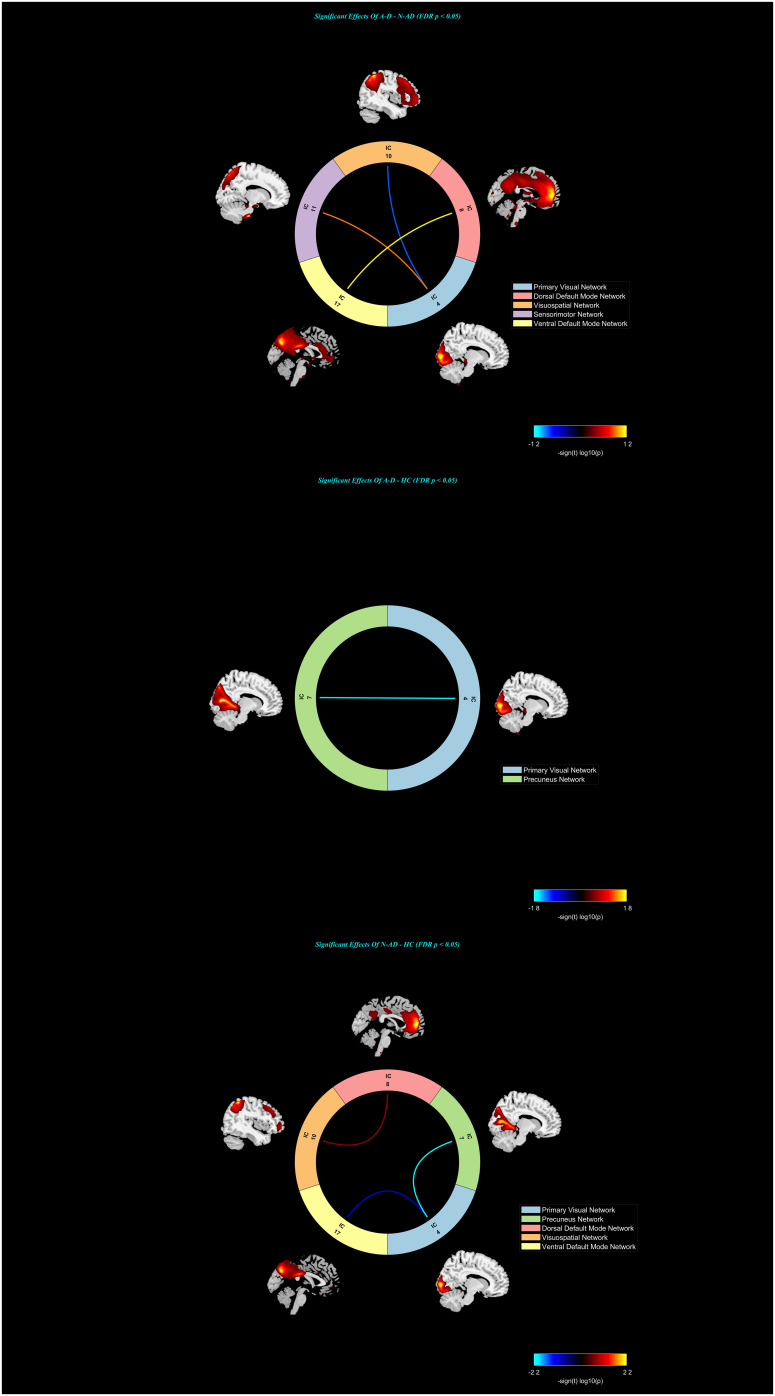
The FNC comparison of the three groups. FNC, functional network connection; IC, independent component; A-D, atypical depression; N-AD, non-atypical depression.

**Figure 3:**
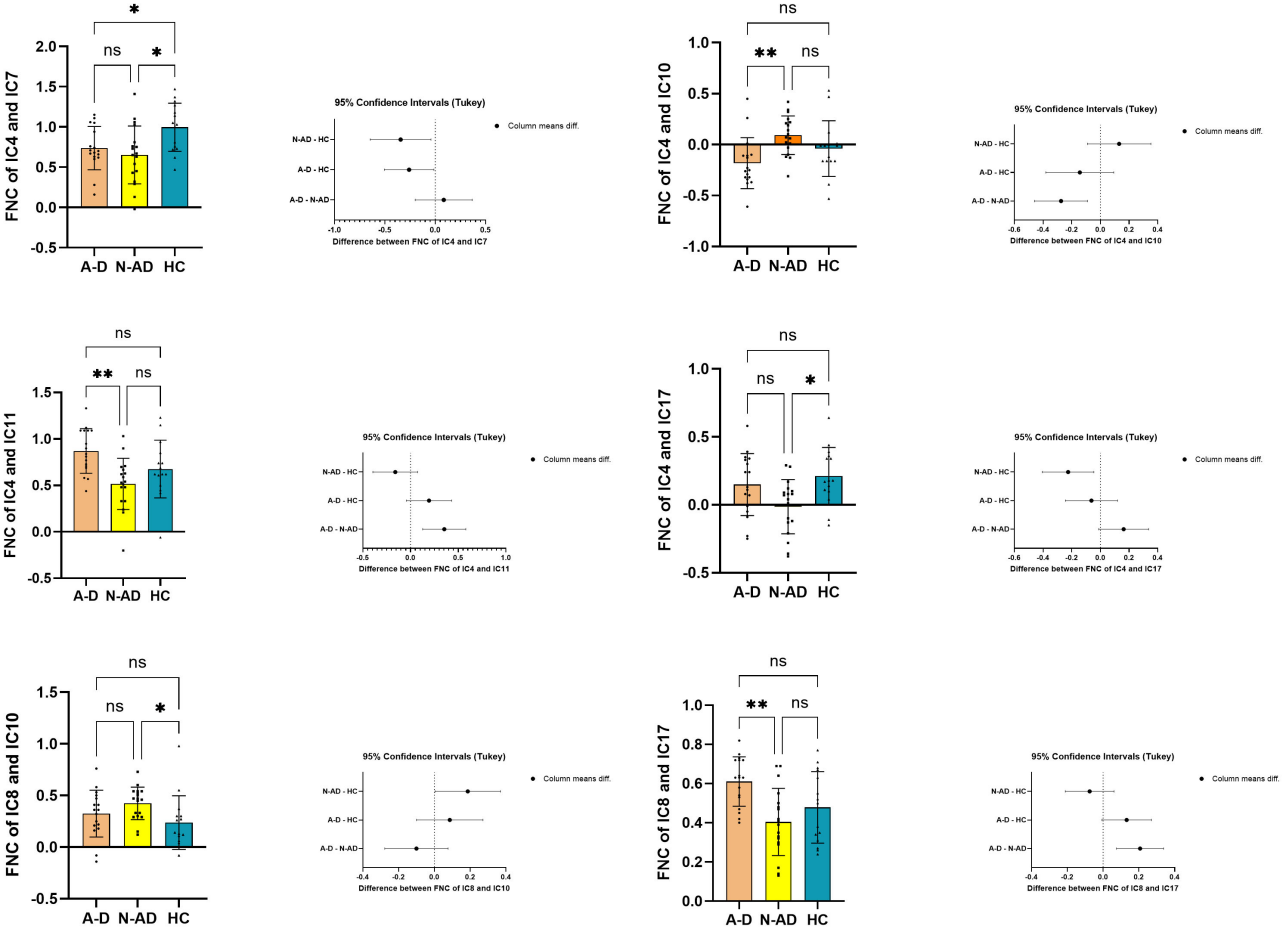
The ANOVA differences of the FNC between the ICs. FNC, functional network connection; HC, healthy control; A-D, atypical depression; N-AD, non-atypical depression; CI, confidence interval; IC, independent component. * *p* < 0.05; ** *p* < 0.01. ns mean nonsignificant.

ANOVA and Tukey’s HSD *post-hoc* analysis were performed on the FNC between the identified components, followed by multiple comparisons correction with the FDR *p* < 0.05. Significant differences were found between the A-D group and the N-AD group. Decreased connectivity was observed between the primary visual network (IC4) and the visuospatial network (IC10) in the A-D group compared to the N-AD group. Increased connectivity was observed between the primary visual network (IC4) and the sensorimotor network (IC11) in the A-D group compared to the N-AD group. Increased connectivity was observed between the ventral default mode network (IC17) and the dorsal default mode network (IC8) in the A-D group compared to the N-AD group. The A-D group showed decreased FNC between the primary visual network (IC4) and precuneus network (IC7) compared to HC. The N-AD group showed decreased FNC between the primary visual network (IC4) and precuneus network (IC7), and primary visual network (IC4) and ventral default mode network (IC17), and increased FNC between the dorsal default mode network (IC8) and visuospatial network (IC10) compared to HC. Cohen’s *f* showed that the FNC between IC8 and IC10 exhibited a moderate effect, and the other results exhibited large effects. Subsequent correlation analysis revealed that both the FNC between IC4 and IC10 and the fALFF value within the right fusiform face area (FFA.R) region of IC4 were correlated with the atypical clinical symptoms in the A-D group. Consequently, subsequent analyses focused exclusively on IC4. See **Figures 2**, **3**, and **Table 2**.

**Table 2:** The ANOVA results of the FNC between the ICs.

FNC	HC	A-D	N-AD	*F* values	*p*-Values	Cohen’s *f*	95% CI
IC4–IC7	0.996 ± 0.300	0.736 ± 0.269	0.652 ± 0.360	5.215	0.009 (A-D *vs* HC: 0.038, N-AD *vs* HC: 0.025)	0.472	A-D *vs* HC: −0.506 to −0.013; N-AD *vs* HC: −0.646 to −0.042
IC4–IC10	−0.039 ± 0.260	−0.182 ± 0.251	0.094 ± 0.190	5.865	0.005 (A-D *vs* N-AD: 0.004)	0.500	A-D *vs* N-AD: −0.461 to −0.088
IC4–IC11	0.668 ± 0.313	0.871 ± 0.242	0.517 ± 0.276	7.164	0.002 (A-D *vs* N-AD: 0.001)	0.553	A-D *vs* N-AD: 0.128 to 0.580
IC4–IC17	0.109 ± 0.230	0.149 ± 0.228	−0.014 ± 0.200	5.023	0.011 (N-AD *vs* HC: 0.011)	0.463	N-AD *vs* HC: −0.405 to −0.045
IC8–IC10	0.237 ± 0.260	0.324 ± 0.227	0.424 ± 0.157	3.886	0.047 (N-AD *vs* HC: 0.045)	0.362	N-AD *vs* HC: 0.004 to 0.369
IC8–IC17	0.481 ± 0.181	0.611 ± 0.127	0.403 ± 0.172	7.425	0.002 (A-D *vs* N-AD: 0.001)	0.562	A-D *vs* N-AD: 0.075 to 0.339

FNC, functional network connectivity; HC, healthy control; A-D, atypical depression; N-AD, non-atypical depression; CI, confidence interval; IC, independent component.

**Figure 4:**
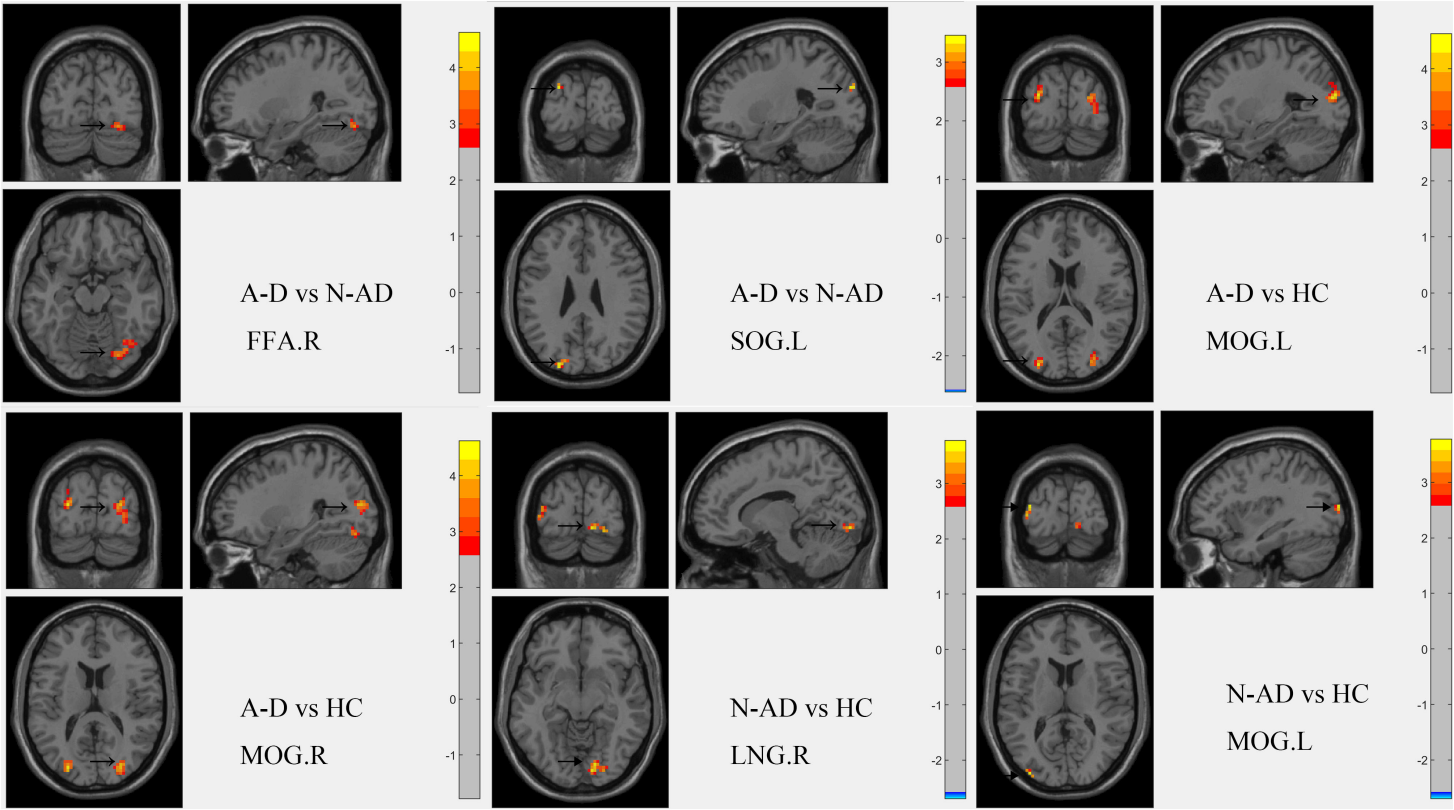
The differential fALFF brain regions between three groups. fALFF, fractional amplitude of low-frequency fluctuations; HC, healthy control; A-D, atypical depression; N-AD, non-atypical depression; FFA.R, right fusiform face area; SOG.L, left superior occipital gyrus; LNG.R, right lingual gyrus; MOG.L/R, left/right middle occipital gyrus.

Comparing the fALFF values of the primary visual network (IC4), we found that the A-D group showed higher fALFF values in the FFA.R and left superior occipital gyrus (SOG.L) than the N-AD group, while showing increased fALFF in the bilateral middle occipital gyrus (MOG) to HC. The N-AD group showed increased fALFF in the left middle occipital gyrus (MOG.L) and right lingual gyrus (LNG.R) compared to HC. The MNI coordinates and voxel sizes are presented in **Table 3**, and the images of the distinct brain regions are shown in **Figure 4**.

**Table 3:** Group differences in MNI coordinates and the voxel size.

Brain region (AAL)	Peak coordinate in MNI space			Cluster size	T value
	X	Y	Z		
A-D *vs* N-AD					
FFA.R	27	−78	−15	85	3.914
SOG.L	−24	−90	27	13	3.459
A-D *vs* HC					
MOG.L	−27	−87	18	43	4.631
MOG.R	27	−84	15	73	4.204
N-AD *vs* HC					
LNG.R	12	−87	−9	39	3.783
MOG.L	−36	−90	12	22	3.587

AAL, Anatomical Automatic Labeling; MNI, Montreal Neurological Institute; FFA.R, right fusiform face area; SOG.L, left superior occipital gyrus; MOG.L/R, left/right middle occipital gyrus; LNG.R, right lingual gyrus; A-D, atypical depression; N-AD, non-atypical depression.

#### Analysis of correlations

**Figure 5:**
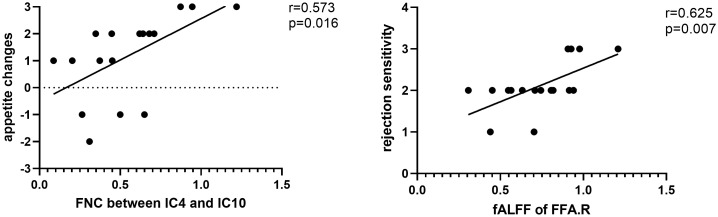
Correlation analysis of the rs-fMRI values in primary visual cortex and the clinical rating scores of A-D symptoms. A-D, atypical depression; FFA.R, right fusiform face area; fALFF, amplitude of low-frequency fluctuations; FNC, functional network connectivity; IC, independent component; IDS-30R, 30-item Inventory of Depressive Symptomatology. Note: the negative values of appetite changes were obtained from the 11th item (appetite decrease) of IDS-30R.

Analysis of the correlation between the A-D clinical symptom scores assessed using the IDS-30R scale in the A-D group and both the FNC between IC4 and IC10 and the fALFF values showing significant between group differences revealed the following key findings: appetite/weight changes showed a significant positive correlation with the FNC between IC4 and IC10 (*r* = 0.573, r^2^ = 0.328, *p* = 0.016). Interpersonal rejection showed a significant positive correlation with the fALFF value in the FFA.R (*r* = 0.625, r^2^ = 0.391, *p* = 0.007). See **Figure 5**.

## Discussion

In our study, we conducted the ICA and fALFF analyses to analyze the functional connectivity between the intrinsic activity in the independent components and their correlation with atypical depressive symptoms. Our main findings were as follows: 1) compared to the N-AD group, the A-D group showed decreased FC between the primary visual network (IC4) and the visuospatial network (IC10) and increased FC between the primary visual network (IC4) and the sensorimotor network (IC11) and between the dorsal default mode network (IC8) and ventral default mode network (IC17). The A-D group showed decreased FC between the primary visual network (IC4) and precuneus network (IC7) compared to HC. The N-AD group showed decreased FC between the primary visual network (IC4) and precuneus network (IC7), and the primary visual network (IC4) and ventral default mode network (IC17), and increased FNC between the dorsal default mode network (IC8) and visuospatial network (IC10) compared to HC. Cohen’s *f* showed that the FC between IC8 and IC10 exhibited a moderate effect, and the other results showed large effects. 2) The A-D group showed higher fALFF values in the FFA.R and SOG.L than the N-AD group while showing increased fALFF in the bilateral MOG compared to HC. The N-AD group showed increased fALFF in the MOG.L and LNG.R compared to HC. 3) The score of changed appetite showed positive correlation with FC between IC4 and IC10, and the scores of interpersonal rejection sensitivity showed positive correlation with the fALFF value of the FFA.R. Aberrant FC within the primary visual network and its inner aberrant function may be correlated with the neural mechanisms of the adolescent A-D clinical symptoms.

Multiple cortical and subcortical brain regions form specialized networks to support visual processing, a critical function for human experience (38). The primary visual network receives input from the eyes and retina and subsequently transmits information to the secondary visual network (39). As the core region of the primary visual network, the primary visual cortex (V1) in humans is commonly studied in relation to the processing of exogenous visual stimuli and is known to be modulated by cognitive processes such as attention, memory, and perceptual learning (40). However, a recent review (41) suggests that V1, traditionally not considered involved in emotion processing, exhibits aberrant functions in MDD. Sanacora et al. (42) reported that individuals with MDD show increased gray matter volume (GMV) in V1 compared to HCs, while the total volume and white matter volume in this region are reduced. This implies that white matter, particularly its intrinsic fiber bundles, which underlie the brain’s structural connectivity, is often reduced in volume, suggesting diminished structural connections between V1 and other brain regions. Furthermore, magnetic resonance spectroscopy (MRS) studies (43) have revealed decreased gamma-aminobutyric acid (GABA) concentrations in the occipital cortex of MDD subjects, indicating a potential shift in the excitatory–inhibitory neurotransmitter balance that may contribute to altered visual cortical function. Another study (44) linked repetitive negative thinking—a core symptom of depression often manifested as rumination—to aberrant connectivity of the primary sensory cortex, including V1. These functional and structural abnormalities may be partly explained by the orientation selectivity of V1 neurons (45), which are highly sensitive to stimulus orientation, unlike their afferent thalamic inputs. Disruption of cortical inhibition can alter this orientation selectivity, and V1 neurons exhibit contrast-invariant orientation tuning and cross-orientation suppression (46). Through these mechanisms, altered inhibition in V1 may affect spontaneous neural activity and neuronal selectivity. From this, we speculate that this may be the cause of abnormal connectivity between the primary visual network and other networks in the brains of MDD patients.

The visuospatial network—involving the bilateral precentral, parietal, and temporal gyri and the cerebellum (47)—is frequently altered in depression, potentially contributing to cognitive deficits such as impaired visuospatial attention (48). In our study, the negative FNC observed between IC4 and IC10 may reflect inverse temporal coupling, suggesting functional differentiation rather than impaired integration. This pattern of anticorrelation is commonly observed between networks with opposing functional roles and may indicate typical network segregation. While visual cues are known to influence appetite through overlapping neural pathways involving reward and mood regulation (49), our interpretation—that the altered FNC may be linked to peripheral dopamine signaling and appetite changes—remains hypothetical and warrants further investigation. Future work should clarify whether these visual and visuospatial networks are involved in reward or appetite processing based on prior mechanistic evidence.

In addition to the visual and visuospatial networks, other networks showed altered connectivity in the A-D group. The sensorimotor network demonstrated increased FNC with the primary visual network, while the ventral and dorsal default mode networks (IC17) showed heightened inner connectivity—consistent with previous reports in depression (50). The default mode network is implicated in self-referential rumination and emotion regulation (51, 52), and its hyperconnectivity has been consistently observed in depressive disorders. Furthermore, regions such as the LNG.R, right middle occipital gyrus (MOG.R), and SOG.L also exhibited functional alterations, in line with prior studies reporting their involvement in emotional processing in MDD (53–57). The aberrant activity in these regions may still contribute to the neurofunctional profile of A-D, even though we found no direct correlation with clinical symptom scores in this cohort. This suggests that their role may be mediated by other factors or require a different paradigm to detect.

Beyond the primary visual networks, we also observed alterations in the fusiform face area (FFA). As a specialized visual region, the FFA supports face perception (57) and shows disrupted connectivity with the prefrontal and limbic regions in depression (58–60). The positive correlation between interpersonal rejection sensitivity and FFA activity in our study may tentatively suggest a role for social perceptual processing in adolescent depression, consistent with models linking FFA function to social cue sensitivity (61, 62). However, this remains an ancillary finding requiring confirmation in studies specifically designed to probe social perception.

Overall, our findings point to a potential association between functional abnormalities in the primary visual and visuospatial networks and core symptoms of A-D. Impaired visual network function may disrupt the analysis and integration of visual information, representing a candidate neuropsychiatric mechanism in A-D. This perspective is partly supported by Liu et al. (63), who demonstrated that rTMS targeting the visual cortex alleviated symptoms in adolescents with major psychiatric disorders. Future studies should employ longitudinal or experimental designs to clarify the directionality and neural mechanisms underlying these network alterations.

This study has several limitations. First, the sample size was very small, so our findings should be considered preliminary and exploratory carefully, requiring validation in future studies with larger, independent cohorts. Second, all MDD patients included in the study underwent pharmacological treatment, which complicates the interpretation of the results. Additionally, due to the heightened risk of negative thoughts leading to behaviors such as self-harm or suicide in individuals with MDD, the administration of medication, while ethically necessary, may introduce confounding effects. Third, the analysis only involved baseline data; the inclusion of post-treatment data would provide more insights into discussing the mechanisms of A-D.

## Data Availability

The raw data supporting the conclusions of this article will be made available by the authors, without undue reservation.
